# A Pilot Study: Changes of Gut Microbiota in Post-surgery Colorectal Cancer Patients

**DOI:** 10.3389/fmicb.2018.02777

**Published:** 2018-11-20

**Authors:** Jing Cong, Hua Zhu, Dong Liu, Tianjun Li, Chuantao Zhang, Jingjuan Zhu, Hongying Lv, Kewei Liu, Chenxing Hao, Zibin Tian, Jianli Zhang, Xiaochun Zhang

**Affiliations:** ^1^Department of Medical Oncology, The Affiliated Hospital of Qingdao University, Qingdao University, Qingdao, China; ^2^Qingdao Cancer Institute, Qingdao, China; ^3^Department of Gastroenterology, The Affiliated Hospital of Qingdao University, Qingdao University, Qingdao, China; ^4^Department of General Surgery, The Affiliated Hospital of Qingdao University, Qingdao University, Qingdao, China

**Keywords:** gut microbiota, colorectal cancer, surgery, high-throughput sequencing, real-time quantitative PCR

## Abstract

Colorectal cancer (CRC) is a growing health problem throughout the world. Strong evidences have supported that gut microbiota can influence tumorigenesis; however, little is known about what happens to gut microbiota following surgical resection. Here, we examined the changes of gut microbiota in CRC patients after the surgical resection. Using the PCoA analysis and dissimilarity tests, the microbial taxonomic compositions and diversities of gut microbiota in post-surgery CRC patients (A1) were significantly different from those in pre-surgery CRC patients (A0) and healthy individuals (H). Compared with A0 and H, the Shannon diversity and Simpson diversity were significantly decreased in A1 (*P* < 0.05). Based on the LEfSe analysis, the relative abundance of phylum *Proteobacteria* in A1 was significantly increased than that in A0 and H. The genus *Klebsiella* in A1 had higher proportions than that in A0 (*P* < 0.05). Individual variation was distinct; however, 90% of CRC patients in A1 had more abundances of *Klebsiella* than A0. The *Klebsiella* in A1 was significantly associated with infectious diseases (*P* < 0.05), revealed by the correlation analysis between differentiated genera and metabolic pathway. The *Klebsiella* (*Proteobacteria, Gammaproteobacteria, Enterobacteriales, Enterobacteriaceae*) in A1 was significantly linked with lymphatic invasion (*P* < 0.05). Furthermore, the PCA of KEGG pathways indicated that gut microbiota with a more scattered distribution in A1 was noticeably different from that in A0 and H. The nodes, the links, and the kinds of phylum in each module in A1 were less than those in A0 and H, indicating that gut microbiota in A1 had a relatively looser ecologcial interaction network. To sum up, this pilot study identified the changes of gut microbiota in post-surgery CRC patients, and highlights future avenues in which the gut microbiota is likely to be of increasing importance in the care of surgical patients.

## Introduction

Colorectal cancer (CRC) is the third leading cause of cancer mortality in the world (Jemal et al., 2011), and is influenced by heredity, diet, lifestyle, gut microbiota, and other factors (Berstad et al., 2015; Sun and Kato, 2016). The human intestinal tract is a nutrient-rich environment housing the largest microbial communities (Zheng et al., 2016). The gut microbiota has garnered great attentions because of its important role in influencing CRC risk by metabolites and immunity in the host (Saleh and Trinchieri, 2011). For example, some bacteria producing hydrogen sulfide, acetaldehyde and secondary bile acids can contribute to the risk of CRC (Huycke and Gaskins, 2004; Bernstein et al., 2009). Simultaneously, some bacteria, including the orders *Clostridiales, Lactobacillales, Bifidobacteriales*, and *Actinomycetales* (Devillard et al., 2007), may reduce CRC risk by producing butyrate and conjugated linoleic acids (Scharlau et al., 2009). Therefore, understanding the role of gut microbiota contributes to improving CRC patients’ care.

Human gut microbiota is considered as an essential “organ,” which plays a key role in providing nourishment, regulating epithelial development, and modulating immunity (Eckburg et al., 2005). In recent years, many researchers have attempted to understand the differences in gut microbiota by comparing the microbial community structures of CRC patients and healthy individuals and identify reliable microbial markers for CRC precursors (Zeller et al., 2014). Previously, it has been reported that the abundances of *Enterococcus faecalis* (Balamurugan et al., 2008) and *Desulfovibrio* sp. (Scanlan et al., 2009) were observably higher in CRC patients than that in healthy individuals, whereas *Bacteroides/Prevotella* levels were significantly lower (Sobhani et al., 2011). Differences were also observed in the overall structure of gut microbiota between CRC patients and healthy individuals (Sobhani et al., 2011). Despite of advances in understanding the connections between gut microbiota and CRC, little is known about how surgery resection influences the gut microbiota. Current studies have indicated that many patients who undergo treatment may experience recurrence and even die within several years (Ryuk et al., 2014). Therefore, identifying valid methods to evaluate post-surgery patients’ condition is vital in reducing mortality and healthcare costs.

Gut microbiota is strongly influenced by the surgical removal of lesions, and influences the intestinal healing, particularly with respect to anastomotic tissues in colorectal surgery (Bachmann et al., 2017). In this pilot study, we analyzed fecal samples of CRC patients and healthy individuals by 16S rRNA gene sequencing and real-time quantitative PCR. First, we used the classical community analysis and statistical tests to compare the gut microbial community structure and composition between CRC patients and healthy individuals. Next, we generated functional discrepancy prediction and molecular ecological network to further examine the differences among them. Finally, we discussed the correlates of gut microbiota and clinical variables for the probabilities to assess whether gut microbiota played a key role in identifying the condition for post-surgery CRC patients.

## Materials and Methods

### Study Design

Our pilot study subjects comprised 10 CRC patients and 11 healthy individuals (Supplementary Figure S1). The CRC patients, aged 34–63 years, were from the affiliated hospital of Qingdao University (Qingdao, China, Table 1 and Supplementary Tables S1, S2). The pilot study selected from untreated CRC patients and excluded those (*N* = 6) who had previously undergone surgery, chemotherapy, radiation, or targeted therapies before samples collection. Fecal samples from these pre-surgery CRC patients were collected prior to a colonoscopy (O’Brien et al., 2013). The lesion location of all the selected CRC patients was in rectum. These CRC patients were treated with palliative surgery or radical surgery, such as Dixon, Miles and Hartmann (Supplementary Table S2). Following up samples were obtained in approximately 1 month after the surgery. The healthy individuals, aged 49–64 years, were selected as controls (Table 1 and Supplementary Table S2). During a routine physical examination, none had any recorded antibiotics usage or gastrointestinal tract disorders within 3 months preceding the sample collection. All of the participants have been local residents of Qingdao city. This pilot study was approved by the Affiliated Hospital of Qingdao University Institutional Review Board, and all pilot study participants signed the informed consent before participation. All fecal samples were collected within 3 h after defecation in the morning. The collected samples from the healthy individuals, pre-surgery CRC patients and post-surgery CRC patients were named by H, A0, and A1, respectively. Fresh fecal samples were put into 5 ml tubes and immediately stored at -80°C until the day of analysis.

**Table 1:** Baseline characteristics in healthy individuals and colorectal cancer patients.

Variable	Healthy individuals	Colorectal cancer patients	*P*-value
Number	11	10	/
Age, year, median (IQR)	60 (49–64)	59 (34–63)	0.386
Sex (Female/Male), n	9/2	4/6	0.051
BMI, median (IQR)	24.1 (21.4–28.2)	25.5 (19.5–31.8)	0.211
Tumor location	/	Rectum	/

### DNA Extraction, Purification, Sequencing and Data Processing

Extraction of bacterial DNA was performed from fecal samples using a QIAamp Fast DNA Stool Mini Kit as previously reported (Wu et al., 2016). The freshly extracted DNA was purified by 0.5% melting point agarose gel followed by phenolchloroform-butanol extraction. The V3-V4 region of the bacterial 16S rRNA gene from each DNA sample was amplified using the bacterial universal primers (forward primer: 5′-ACTCCTACGGGRSGCAGCAG-3′; reverse primer: 5′-GGACTACVVGGGTATCTAATC-3′). PCR amplification was performed in a 30 μl reaction, containing 15 μl of 2 × KAPA HiFi Hotstart ReadyMix, 1 μl of each primer (forward and reverse primer), 10 ng of template DNA, and the remaining volume of ddH_2_O. The reaction mixtures were denatured at 95°C for 1 min; followed by 12 cycles of 98°C for 15 s, 72°C for 10 s, 94°C for 20 s, 65°C for 10 s and 72°C for 10 s; then 11 cycles of 94°C for 20 s, 58°C for 30 s, 72°C for 30 s; and a final extension at 72°C for 150 s. The PCR amplification products were purified with an AxyPrep DNA Gel Extraction Kit (Axygen, United States), eluted in 30 μl water, and aliquoted into three PCR tubes. DNA quality and quantity were assessed by the ratios of 260/280 nm and 260/230 nm, and final DNA contents were quantified with a Qubit^®^ dsDNA HS Assay Kit (Invitrogen, United States). Finally, we used bacterial DNA amplicons from each fecal sample for 2 × 250 bp paired-end sequencing based on the Illumina Hiseq 2500.

Raw sequences were separated into samples by barcodes using the Galaxy Illumina sequencing pipeline^1^. Adapters, ambiguous and low-quality reads (“N”) were trimmed by Btrim (Kong, 2011). Forward and reverse reads were incorporated into a whole sequence by FLASH (Magoč and Salzberg, 2011). After quality control of the raw data, the clean reads were clustered into operational taxonomic units (OTUs) at 97% similarity level by using UCLUST (Edgar, 2010). Each OTU was considered to represent a species (Deng et al., 2012). Rarefaction analysis was performed using the original detected OTUs (Supplementary Figure S2). The ribosomal database project (RDP) classifier was used to determine the taxonomic assignment (Wang et al., 2007). Random resampling was conducted on 48,360 sequences per fecal sample.

### Real-Time Quantitative PCR

Three specific primers were used for real-time quantitative PCR (qPCR), including 16S rRNA universal primer for bacteria, primer for *Fusobacterium nucleatum* (Castellarin et al., 2012), primer for *Klebsiella pneumonia* (Sun et al., 2010) (Supplementary Table S3). The primers were synthesized at Shanghai Sangon Company (China). The PCR program was as follows: 95°C for 3 min; 35 cycles of 94°C for 30 s, 57°C for 30 s, and 72°C for 30 s; 72°C for 8 min. The PCR products were purified with gel extraction kit (Sangon SK8131), quantified using Micro-spectrophotometer SMA4000 (Merinton), and used to construct standard curves. The copy number of PCR products was calculated based on the formula: Copy number/μL = 6.02 × 10^14^ × C/(*M* × *W*). The C (unit, ng/μL) represents the concentration of PCR products, *M* (unit, bp) represents the length of PCR products, and *W* (660 Da/bp) represents the constant. The PCR products were diluted from 10^7^ to 10^10^ copies/μL and amplified to construct standard curves. The real-time qPCR was run with LightCycler480 II (Roche, German). The reaction mixture contained 5 μL SybrGreen qPCR Master Mix (Roche, German), 1 μL of template DNA, and 0.2 μL forward/reverse primer (10 μM), and ddH2O was added to reach a total volume of 10 μL. The real-time qPCR program was as follows: 95°C for 3 min; 45 cycles of 95°C for 15 s, 57°C for 20 s, and 72°C for 30 s. The melting curves for the amplicons were measured while monitoring fluorescence. The amplification efficiencies of three primers were between 80 and 110%, and the melting curves all showed a single peak (Supplementary Figure S3), indicating that the results were credible. The copies of each sample based on 16S rRNA universal primer were considered as the bacterial biomass per gram. The following formula was used to calculate the relative abundance: Relative abundance = Ci/C0 × 100%, where Ci represents the copies of the species and C0 represents the bacterial biomass (Xiao et al., 2018).

### Network Analysis

Gut microbial ecological networks were constructed and analyzed by random matrix theory (RMT) methods by the online MENA pipeline^2^. OTUs detected in less than 70% from each group were removed to ensure reliable correlations. For comparisons with different networks, the same cutoff of 0.77 was applied to construct ecological networks for gut microbial communities. Each ecological network was separated into modules by the fast greedy modularity optimization to characterize the modularity property. Furthermore, a network developed from OTU abundance data represented the ecological co-occurrence (links) of different OTU markers (nodes) in a microbial community, and different nodes played distinct roles (Guimerà et al., 2007).

### Statistical Analysis

The common OTUs mean that the OTUs present in three groups (A1, A0 and H). Principal coordinate analysis (PCoA) was used to identify overall gut microbial composition between CRC patients and healthy individuals based on Bray-Curtis dissimilarity index. Principal components analysis (PCA) was used to determine the changes of KEGG pathways between CRC patients and healthy individuals. Alpha diversity was calculated using the observed species (richness), phylogenetic diversity, Shannon index and Simpson index. The significant differences referred to the multiple response permutation procedure (MRPP) algorithms and analysis of similarity (Anosim). Significant *P*-values associated with microbial clades and functions were identified by Linear Discriminant Analysis with Effect Size (LEfSe). Characteristics with an LDA score cut-off of 2.0 were considered as being different. Community analysis and differential abundance of OTUs were performed using the STAMP 2.0.8 (Parks et al., 2014). According to the Kyoto Encyclopedia of Genes and Genomes (KEGG) orthology, functional profiling of microbial communities was predicted using Phylogenetic Investigation of Communities by Reconstruction of Unobserved States (PICRUSt) (Langille et al., 2013). Gut microbial metabolic and other pathway differences were predicted by the correlations between the PICRUSt-generated functional profiles and STAMP-generated genus level bacterial abundance. Mantel test was used to evaluate the linkages between gut microbial structure and environmental attributes. The R software package (v3.4.1) was used for all statistical analysis, except for two-tailed unpaired *t*-tests by Microsoft Excel 2010, and Analysis of variance (ANOVA) and Pearson correlation by IBM SPSS statistic 19.0 to determine the significance of the differences and the clinical correlates.

## Results

### Taxonomic Composition and Diversity of Gut Microbiota

**FIGURE 1:**
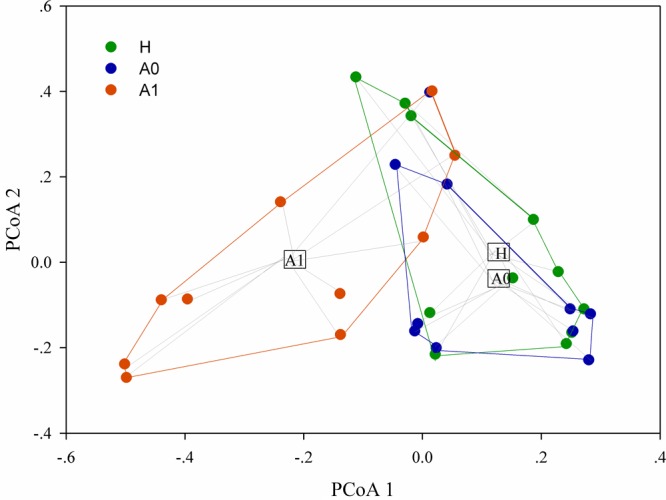
Principal coordinates analysis (PCoA) ordination (operational taxonomic units = 97% 16S rRNA sequence similarity) showing distinctly different microbial composition between CRC patients and healthy individuals based on the Bray-Curtis dissimilarity matrix.

A total of 1,819,210 quality-filtered 16S rRNA gene sequences were acquired from 31 samples, with an average of 58,684 ± 2602 reads per sample (Supplementary Table S4). A total of 648 OTUs were generated at the 97% similarity level, with an average of 189 ± 60 OTUs per sample (Supplementary Table S4). We compared the microbial alpha diversity (richness, phylogenetic diversity, Shannon diversity and Simpson diversity) between CRC patients and healthy individuals (Table 2). The results demonstrated that the Shannon diversity and Simpson diversity were significantly decreased in A1 compared with the A0 and H (*P* < 0.05, Table 2). However, no statistically significant differences were identified in the richness and phylogenetic diversity for CRC patients and healthy individuals (*P* > 0.05, Table 2). The dissimilarity tests showed that A1 was significantly different from A0 and H based on the multiple response permutation procedure (MRPP) algorithms and analysis of similarity (ANOSIM) (*P* < 0.05, Supplementary Table S5). PCoA based on Bray-Curtis dissimilarity index revealed overall gut microbial composition in CRC patients was well separated from each other, but partly overlapping with healthy individuals (Figure 1). Furthermore, A1 presented a more scattered distribution and had a distance from the A0 and H (Figure 1). Therefore, A1 had significantly different community structure with A0 and H.

**Table 2:** Comparison of alpha diversity indices of gut microbiota between the healthy volunteers (H) and CRC patients before and after surgery (A0 and A1).

Group	Richness	Phylogenetic diversity	Shannon diversity	Simpson diversity
A0	(199 ± 56)a	(15.10 ± 4.00)a	(4.63 ± 0.91)a	(0.90 ± 0.08)a
A1	(166 ± 77)a	(14.14 ± 5.25)a	(3.40 ± 1.27)b	(0.76 ± 0.23)b
H	(187 ± 41)a	(14.31 ± 2.44)a	(4.34 ± 0.91)a	(0.88 ± 0.08)a

*Values are shown as mean ± standard deviation. Richness means the detected gene/sequence numbers. Significant differences among different groups are indicated by alphabetic letters, *P* < 0.05. The ‘a’ means there are no significance differences among groups. The ‘b’ means the group is significantly different from other two groups.*

### Taxonomy-Based Comparisons of the Gut Microbiota

**FIGURE 2:**
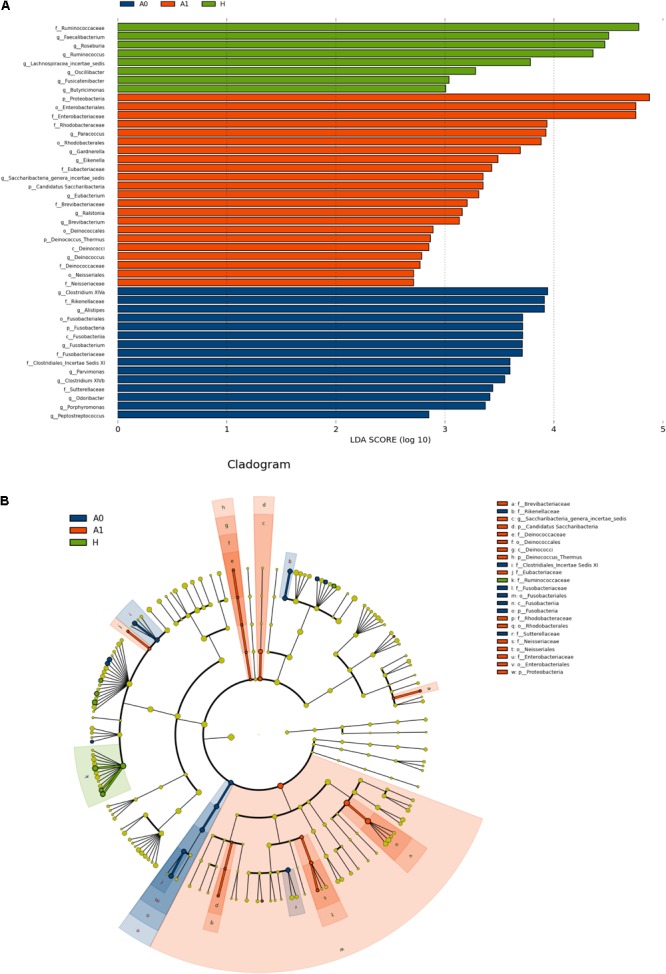
Microbial biomarkers among healthy volunteers (H) and CRC patients (A0 and A1). **(A)** LEfSe analysis shows differentially abundant taxa as biomarkers using Kruskal–Wallis test (*P* < 0.05) with LDA score > 2.0. **(B)** Cladogram representation of the differentially abundant taxa. The root of the cladogram represents the domain bacteria. The size of each node represents their relative abundance. No significantly different taxa are labeled by yellow. Significant different taxa are labeled by following the color of each group.

The gut microbial taxa and their relative abundance were significantly different among H, A0, and A1. At the phylum level, H was mainly characterized by *Firmicutes* and *Bacteroidetes*, whereas A0 and A1 had a very complicated community composition, especially for A1 (Supplementary Figure S4). Compared with H, the relative abundance of *Proteobacteria* was significantly increased by 12.90%, and *Bacteroidetes* was significantly decreased by 23.06% in A1 (*P* < 0.05, Supplementary Table S6). At the genus level, the members of *Faecalibacterium, Roseburia, Ruminococcus*, and *Lachnospiracea_incertae_sedis* were significantly lower in A1 than those in H (*P* < 0.05, Supplementary Figure S5 and Supplementary Table S7). To identify gut microbial responses associated with surgery at the taxonomical level, we determined microbial clade differences using LEfSe analysis (Figure 2). At the phylum level, higher proportions of *Proteobacteria* were observed in A1 than that in A0 and H (Figure 2A). At the genus level, greater proportions of *Klebsiella* were detected in A1 than that in A0 (Supplementary Figure S6). The genus *Fusobacteria* was significantly enriched in A0 than that in A1 and H (Figure 2B). The members of *Clostridium XlVa, Fusobacterium, Parvimonas*, and *Peptostreptococcus* were more abundant on A0 than that on A1 and H (Figure 2A).

Sequencing data suggested that gut microbiota made changes in response to surgery, and we further used real-time qPCR to help validate changes observed and detected with 16S sequencing data. We selected *Fusobacterium nucleatum* and *Klebsiella pneumoniae* to examine the variations, which frequently present in CRC patients (Supplementary Figure S7). The results showed that the relative abundance of *Fusobacterium nucleatum* in A0 was significantly higher than that in H (*P* < 0.05). However, the relative abundance of *Klebsiella pneumoniae* was not significantly changed. In addition, individual differences were evident showed in Supplementary Figure S8. Notably, the relative abundance of *Fusobacterium* increased markedly in 8/10 patients of A0 compared with A1. The members of *Klebsiella* were distinctly higher in 9/10 patients of A1 than that in A0.

### Functional and Metabolic Discrepancy of the Gut Microbiota

**FIGURE 3:**
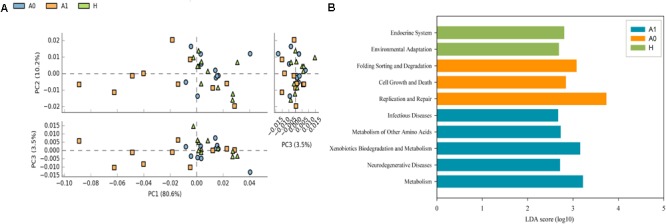
Functional and metabolic discrepancy of the gut microbiota between CRC patients and healthy individuals. **(A)** Principal component analysis (PCA) plot of the KEGG pathway (L2) shows that the post-surgery CRC patients were noticeably different from the pre-surgery CRC patients and healthy individuals based on the STAMP analysis. Characteristics with an LDA score cut-off of 2.0 were considered as being different. The LDA scores (log10) > 2 are listed; **(B)** Discriminatory functional pathways (KEGG L2) shows the significantly different between CRC patients and healthy individuals based on the LDA score using the LEfSe analysis.

**FIGURE 4:**
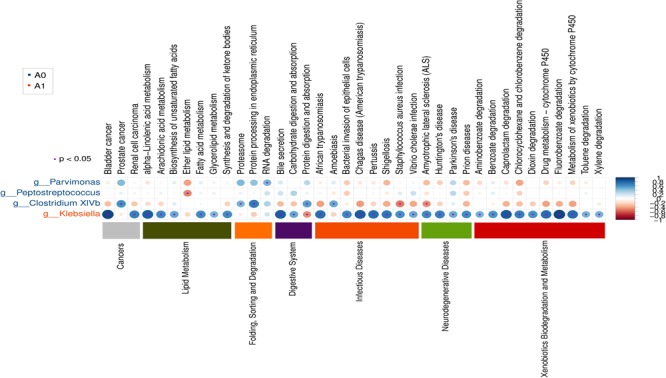
Gut microbial metabolic and other pathway differences in pre-surgery CRC patients (A0) and post-surgery CRC patients (A1). Correlations between the PICRUSt-generated functional profiles and STAMP-generated genus level bacterial abundance are calculated and plotted.

Further studies were required to understand the dynamics of gut microbiota following surgical treatment to evaluate the role of microbiota. The PCA of KEGG pathways indicated that A1 was notably different from A0 and H, which had a more scattered distribution based on the STAMP analysis (Figure 3A). In terms of KEGG pathways (L2, Figure 3B), the functions of ‘replication and repair,’ ‘folding, sorting and degradation,’ and ‘cell growth and death,’ which belong to the first level ‘Genetic information processing’ and ‘Cellular processes,’ respectively, were significantly enriched in A0 (*P* < 0.05) compared with H and A1 based on the LEfSe analysis. The results indicated that gut microbiota in pre-surgery patients was enriched in more conservative housekeeping functions. The ‘infectious diseases,’ ‘xenobiotics biodegradation and metabolism,’ ‘metabolism of other amino acids,’ ‘neurodegenerative diseases,’ and ‘metabolism’ were significant function hallmarks of gut microbiota in A1. Furthermore, we found that the genus *Klebsiella* in A1 was significantly and closely associated with infectious diseases, such as bacterial invasion of epithelial cells and *Staphylococcus aureus* infection (*P* < 0.05, Figure 4).

### Modularity Analysis in Gut Microbiota

**FIGURE 5:**
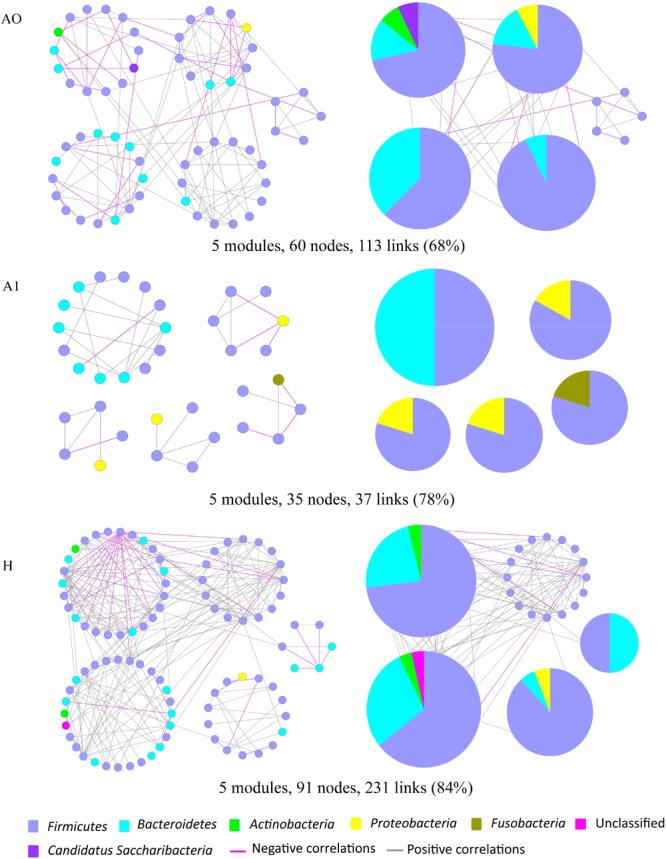
Highly connected modules of gut microbial networks within CRC patients before and after surgery (A0, A1) and healthy individuals (H). The colors of nodes indicate different major phyla; pie charts represent the composition of modules with ≥ 2 phyla. A pink link indicates negative correlations between two individual nodes, whereas a gray link indicates positive correlations. The percentage in parentheses indicates the ratio of positive correlations.

Microbes rarely live in isolation, but instead interact in complex ecological networks. To identify the assemblages that potentially interact within the intestinal tract, we focused on representative networks from CRC patients and healthy individuals. We selected more than five nodes to construct the modules and visualized the phylogeny for modules with at least two kinds of phyla (Figure 5). Overall, OTU tended to co-occur (positive correlations, gray lines) rather than co-exclude (negative correlations, pink lines). However, there were more negative correlations between gut microbes in A0 than that in A1 and H (Figure 5). The modules in A1 became smaller and less connected, which had fewer nodes and links (35, 37) than that in A0 (60, 113) and in H (91, 231). Furthermore, the kinds of phylum in each module were only two in A1, which were less than that in A0 and H.

### Correlates of Gut Microbiota and Clinical Variables

Based on the mantel test, we explored the clinical correlates of gut microbiota and patients age, sex, BMI, and bowel treatment. The results showed that the whole gut microbiota and key phyla had no significant correlations with these clinical variables (Supplementary Table S8). Then, we analyzed the histopathological correlates of gut microbiota and tumor stage, grade of tumor differentiation, lymphatic invasion, perineural invasion, number of metastasis lymph nodes and tumor markers (CEA and CA199) (Supplementary Table S9). We found that the *Fusobacterium* (*Fusobacteria, Fusobacteriia, Fusobacteriales, Fusobacteriaceae*) in A0 and *Klebsiella* (*Proteobacteria, Gammaproteobacteria, Enterobacteriales, Enterobacteriaceae*) in A1 were significantly correlated with lymphatic invasion (*P* < 0.05). The *Fusobacterium* (*Fusobacteria, Fusobacteriia, Fusobacteriales, Fusobacteriaceae*) in A0 was significantly correlated with CA199 (*P* < 0.05).

## Discussion

The gut microbiota interacts extensively with the host by the co-metabolism of substrates and metabolic exchanges to maintain a healthy status and normal functions of the body (Nicholson et al., 2005). Previous studies have highlighted the significance of gut microbiota in the progression of intestinal diseases such as Crohn’s disease, ulcerative colitis (Willing et al., 2010), celiac disease in children (Nadal et al., 2007), allergic inflammation in infants (Kalliomäki and Isolauri, 2003), and CRC (Bachmann et al., 2017). In this pilot study, we focused on exploring the feedback of gut microbiota of CRC patients in response to the surgical removal of lesions.

The gut microbial community composition and diversity in post-surgery CRC patients significantly differ from that in pre-surgery CRC patients and healthy individuals (*P* < 0.05, Figure 1, Table 2, and Supplementary Table S5). However, the gut microbial alpha diversity in A0 was not significantly different from that in H (Table 2). The findings may be explained by previous report that the fecal microbiota only partially reflected mucosal microbiota in CRC (Flemer et al., 2017a). The alpha diversity in A1 was significantly lower than that in H (*P* < 0.05). High diversity is always linked to health and temporal stability (Flores et al., 2014). Conversely, a relative lack of diversity is often observed in the gut microbiota of CRC patients (Gao et al., 2015). Antibiotics use causes a dramatic reduction in the diversity of gut microbiota (Dethlefsen and Relman, 2011), which was similar to our results that the gut microbial diversity was significantly decreased in post-surgery CRC patients, potentially weakening the community’s ability to resist pathogens.

Flemer et al. (2017b) had previously confirmed that the microbiota of CRC patients differed from that of controls (Flemer et al., 2017b), and similar results had been found in our study that gut microbial taxa and their relative abundance of CRC patients, especially for post-surgery CRC patients, significantly differed from that of healthy individuals (*P* < 0.05). Generally, the gut microbiota in healthy individuals is dominated by the phyla *Firmicutes* and *Bacteroidetes* (Consortium, 2012), whereas *Proteobacteria, Actinobacteria, Verrucomicrobia*, and *Fusobacteria* are less abundant (Bäckhed et al., 2005). The relative abundance of phylum *Proteobacteria* was significantly higher in A1 than that in A0 and H (*P* < 0.05, Figure 2A and Supplementary Table S6). Imbalanced gut microbiota often results from a sustained increase in *Proteobacteria*, and human gut microbiota normally contains a minor proportion of this phylum (Shin et al., 2015). A bloom of *Proteobacteria* in the gut often reflects an unstable structure of the gut microbial community, which was often observed in the disease states (Morgan et al., 2012). *Proteobacteria* was often associated with dysbiosis and was a potential diagnostic criterion for disease (Shin et al., 2015). The relative abundance of phylum *Fusobacteria* was significantly increased in A0 than that in A1 and H (*P* < 0.05, Figure 2B). At the genus level, *Klebsiella* played more significantly key roles in A1 than that in A0 (*P* < 0.05, Supplementary Figure S6). However, the relative abundance of *Klebsiella pneumoniae* was not significantly changed based on the real-time qPCR (Supplementary Figure S7). The reasons were probably ascribed to the small sample size, followed by the low abundance in sample itself, DNA degradation of human feces, the sensitivity and specificity of primer, reaction condition of qPCR. In addition, *Clostridium XlVa, Parvimonas, Peptostreptococcus*, and *Fusobacterium* played significantly important roles in A0 (*P* < 0.05, Figure 2A). *Parvimonas* was frequently and significantly increased in stools from CRC patients (Shah et al., 2017). *Peptostreptococcus* played a key role in the dysbiosis of mucosa-associated microbiota in CRC patients (Shah et al., 2017). It was reported that *Fusobacterium* species led the development of CRC (Kostic et al., 2012). The qPCR results also showed that *Fusobacterium nucleatum* in A0 was significantly higher than that in H (*P* < 0.05, Supplementary Figure S7). *Fusobacterium nucleatum* infection is prevalent in human CRC (Castellarin et al., 2012). Furthermore, we found that *Fusobacterium* (*Fusobacteria, Fusobacteriia, Fusobacteriales, Fusobacteriaceae*) in A0 was significantly correlated with lymphatic invasion and tumor marker CA199 (*P* < 0.05, Supplementary Table S9), similar to Castellarin et al. (2012) results that indicated that high relative abundance of *Fusobacterium* was more likely to have regional lymph node metastases. Therefore, gut microbiota from fecal samples was increasingly possible to be considered as potential diagnostic biomarkers of dysbiosis for CRC patients (Zackular et al., 2014).

Potential function and metabolism of gut microbiota in CRC patients were greatly changed in response to the surgery. Although great differences were observed in the taxonomic composition of gut microbiota in different individuals, the metabolic pathways are considerably more consistent across people (Consortium, 2012). A healthy microbiota may contain specific microbial combinations, metabolic modules, and regulatory pathways that together maintain a stable host-associated ecology (Martiny et al., 2015). The housekeeping functions are necessary for all microbial life, such as ‘replication and repair’ and ‘cell growth and death’ (Consortium, 2012). Our results demonstrated that the housekeeping functions were significantly associated with gut microbiota in A0, whereas the gut microbiota in A1 was more closely involved with the functions of infectious diseases and xenobiotics biodegradation and metabolism (Figure 3). The *Klebsiella* in A1 was determined to be closely associated with infectious diseases (Figure 4). *Klebsiella* is an opportunistic pathogen routinely found in human gut that causes diarrhea, pneumonia, and bloodstream infections (Yan et al., 2017). It was reported that overgrowth of *Klebsiella* often foreshadowed gut flora dysbiosis (Garrett et al., 2010) and markedly increased the rates of treatment failure and death (Yan et al., 2017). Furthermore, *Klebsiella* (*Proteobacteria, Gammaproteobacteria, Enterobacteriales, Enterobacteriaceae*) in A1 was significantly correlated with lymphatic invasion (*P* < 0.05, Supplementary Table S9). Therefore, the gut microbiota in post-surgery CRC patients maybe presented a weaker robustness when random and specific perturbations influence its functional stability.

Molecular ecological networks of gut microbiota in CRC patients were also greatly changed in response to the surgery. In network biology, a group of microbial species strongly interacting with one another constructs a module, which may reflect physical contact, divergent selection, functional association, and/or the phylogenetic clustering of closely related species (Olesen et al., 2007). Considering the characteristic of a smaller and looser network module in A1 (Figure 5), it implied that A1 had a weaker coupling between gut microbes than A0 and H. This could partially be explained by some sharply growing pathogenic bacterium, such as the phylum *Proteobacteria*, influencing the microbial community structure (Figure 2 and Supplementary Table S6). Therefore, the gut microbiota in post-surgery patients probably had a higher sensitivity in response to external changes.

The age, sex, BMI, bowel treatment and diet were considered as the important influencing factors in changing the gut microbiota of CRC patients. However, we found that there were no significant correlations between these factors (age, sex, BMI, and bowel treatment) and gut microbiota based on the mantel test (Supplementary Table S8). Comprehensive information on microbial species across a great number of samples is essential in identifying the changes among microbial communities (Barberán et al., 2012). Sample sets should ideally be ample to achieve sufficient variability (Barberán et al., 2012). However, the number of patients in the treatment group was relatively small and individual differences were evident (Supplementary Figure S8). Therefore, there was not statistical power to adequately examine these relationships between these factors (age, sex, BMI, and bowel treatment) and gut microbiota. Therefore, we could not scale the results to all the situations with only a few samples. In addition, samples from the post-surgery patients were also influenced by the colonoscopy. More attentions should be paid on these correlative factors in the future. Yet, this pilot study would provide better understanding of the responses of gut microbiota to the surgical removal of lesions for CRC patients (Supplementary Figure S9). The gut microbiota probably plays a key role in identifying the condition for post-surgery CRC patients. Additional sampling efforts, colonoscopy effects, and diet records combined with clinical follow-up are required to further obtain unique insight into gut microbial changes in post-surgery CRC patients to predict disease states and develop therapies to correct dysbiosis.

## Conclusion

In summary, this pilot study explored the changes of gut microbiota in CRC patients following the surgery. The gut microbial taxonomic compositions in post-surgery CRC patients were significantly different from those in pre-surgery CRC patients and healthy individuals. The gut microbiota in post-surgery CRC patients had a significantly lower alpha diversity and a looser ecological interaction network. Most post-surgery CRC patients had more abundances of *Klebsiella*. The *Klebsiella* in post-surgery CRC patients was significantly associated with lymphatic invasion. These results indicated that gut microbiota was probably considered to be the valuable biomarkers in evaluating the condition of post-surgery CRC patients. More attentions should be paid to advance our understanding of the role of gut microbiota in recovering the intestinal health of post-surgery CRC patients.

## Availability of Data and Material

Sequencing data are accessible in NCBI SRA database with Accession No. SRP133809 (https://www.ncbi.nlm.nih.gov/sra/SRP 133809).

## Ethics Statement

The Affiliated Hospital of Qingdao University Institutional Review Board approved this study, and all study subjects provided informed consent.

## Author Contributions

All authors were involved in the design of the study. DL, HZ, and CH collected fecal samples. JjZ, TL, CZ, HL, KL, ZT, and JlZ analyzed the data. All authors interpreted the data. JC participated in all the work including writing the manuscript. All authors reviewed and revised the manuscript and read and approved the final manuscript.

## Conflict of Interest Statement

The authors declare that the research was conducted in the absence of any commercial or financial relationships that could be construed as a potential conflict of interest.

## Footnotes

1 http://zhoulab5.rccc.ou.edu:8080/

2 http://ieg2.ou.edu/MENA/

## References

[B1] Bachmann R. Leonard D. Delzenne N. Kartheuser A. Cani P. D. (2017). Novel insight into the role of microbiota in colorectal surgery. *Gut* 66 739–749. 10.1136/gutjnl-2016-312569 28153961

[B2] Bäckhed F. Ley R. E. Sonnenburg J. L. Peterson D. A. Gordon J. I. (2005). Host-bacterial mutualism in the human intestine. *Science* 307 1915–1920. 10.1126/science.1104816 15790844

[B3] Balamurugan R. Rajendiran E. George S. Samuel G. V. Ramakrishna B. S. (2008). Real-time polymerase chain reaction quantification of specific butyrate-producing bacteria, *Desulfovibrio* and *Enterococcus faecalis* in the feces of patients with colorectal cancer. *J. Gastroenterol. Hepatol.* 23 1298–1303. 10.1111/j.1440-1746.2008.05490.x 18624900

[B4] Barberán A. Bates S. T. Casamayor E. O. Fierer N. (2012). Using network analysis to explore co-occurrence patterns in soil microbial communities. *ISME J.* 6 343–351. 10.1038/ismej.2011.119 21900968 PMC3260507

[B5] Bernstein H. Bernstein C. Payne C. M. Dvorak K. (2009). Bile acids as endogenous etiologic agents in gastrointestinal cancer. *World J. Gastroenterol.* 15 3329–3340. 10.3748/wjg.15.3329 19610133 PMC2712893

[B6] Berstad P. Løberg M. Larsen I. K. Kalager M. Holme Ø Botteri E. (2015). Long-term lifestyle changes after colorectal cancer screening: randomized controlled trial. *Gut* 64 1268–1276. 10.1136/gutjnl-2014-307376 25183203

[B7] Castellarin M. Warren R. L. Freeman J. D. Dreolini L. Krzywinski M. Strauss J. (2012). *Fusobacterium nucleatum* infection is prevalent in human colorectal carcinoma. *Genome Res.* 22 299–306. 10.1101/gr.126516.111 22009989 PMC3266037

[B8] Consortium H. M. P. (2012). Structure, function and diversity of the healthy human microbiome. *Nature* 486 207–214. 10.1038/nature11234 22699609 PMC3564958

[B9] Deng Y. Jiang Y. H. Yang Y. He Z. Luo F. Zhou J. (2012). Molecular ecological network analyses. *BMC Bioinformatics* 13:113. 10.1186/1471-2105-13-113 22646978 PMC3428680

[B10] Dethlefsen L. Relman D. A. (2011). Incomplete recovery and individualized responses of the human distal gut microbiota to repeated antibiotic perturbation. *Proc. Natl. Acad. Sci. U.S.A.* 108(Suppl. 1) 4554–4561. 10.1073/pnas.1000087107 20847294 PMC3063582

[B11] Devillard E. Mcintosh F. M. Duncan S. H. Wallace R. J. (2007). Metabolism of linoleic acid by human gut bacteria: different routes for biosynthesis of conjugated linoleic acid. *J. Bacteriol.* 189 2566–2570. 10.1128/JB.01359-06 17209019 PMC1899373

[B12] Eckburg P. B. Bik E. M. Bernstein C. N. Purdom E. Dethlefsen L. Sargent M. (2005). Diversity of the human intestinal microbial flora. *Science* 308 1635–1638. 10.1126/science.1110591 15831718 PMC1395357

[B13] Edgar R. C. (2010). Search and clustering orders of magnitude faster than BLAST. *Bioinformatics* 26 2460–2461. 10.1093/bioinformatics/btq461 20709691

[B14] Flemer B. Lynch D. B. Brown J. M. R. Jeffery I. B. Ryan F. J. Claesson M. J. (2017a). Original article: tumour-associated and non-tumour-associated microbiota in colorectal cancer. *Gut* 66 633–643. 10.1136/gutjnl-2015-309595 26992426 PMC5529966

[B15] Flemer B. Lynch D. B. Brown J. M. R. Jeffery I. B. Ryan F. J. Claesson M. J. (2017b). Tumour-associated and non-tumour-associated microbiota in colorectal cancer. *Gut* 66 633–643. 10.1136/gutjnl-2015-309595 26992426 PMC5529966

[B16] Flores G. E. Caporaso J. G. Henley J. B. Rideout J. R. Domogala D. Chase J. (2014). Temporal variability is a personalized feature of the human microbiome. *Genome Biol.* 15 1–13. 10.1186/s13059-014-0531-y 25517225 PMC4252997

[B17] Gao Z. Guo B. Gao R. Zhu Q. Qin H. (2015). Microbiota disbiosis is associated with colorectal cancer. *Front. Microbiol.* 6:20 10.3389/fmicb.2015.00020 PMC4313696 25699023

[B18] Garrett W. S. Gallini C. A. Yatsunenko T. Michaud M. Dubois A. Delaney M. L. (2010). *Enterobacteriaceae* act in concert with the gut microbiota to induce spontaneous and maternally transmitted colitis. *Cell Host Microbe* 8 292–300. 10.1016/j.chom.2010.08.004 20833380 PMC2952357

[B19] Guimerà R. Sales-Pardo M. Amaral L. A. N. (2007). Classes of complex networks defined by role-to-role connectivity profiles. *Nat. Phys.* 3 63–69. 10.1038/nphys489 18618010 PMC2447920

[B20] Huycke M. M. Gaskins H. R. (2004). Commensal bacteria, redox stress, and colorectal cancer: mechanisms and models. *Exp. Biol. Med.* 229 586–597. 10.1177/153537020422900702 15229352

[B21] Jemal A. Bray F. Center M. M. Ferlay J. Ward E. Forman D. (2011). Global cancer statistics. *Cancer J. Clin.* 61 69–90. 10.3322/caac.20107 21296855

[B22] Kalliomäki M. Isolauri E. (2003). Role of intestinal flora in the development of allergy. *Curr. Opin. Allergy Clin. Immunol.* 3 15–20. 10.1097/00130832-200302000-00003 12582309

[B23] Kong Y. (2011). Btrim: a fast, lightweight adapter and quality trimming program for next-generation sequencing technologies. *Genomics* 98 152–153. 10.1016/j.ygeno.2011.05.009 21651976

[B24] Kostic A. D. Gevers D. Pedamallu C. S. Michaud M. Duke F. Earl A. M. (2012). Genomic analysis identifies association of Fusobacterium with colorectal carcinoma. *Genome Res.* 22 292–298. 10.1101/gr.126573.111 22009990 PMC3266036

[B25] Langille M. G. Zaneveld J. Caporaso J. G. Mcdonald D. Knights D. Reyes J. A. (2013). Predictive functional profiling of microbial communities using 16S rRNA marker gene sequences. *Nat. Biotechnol.* 31 814–821. 10.1038/nbt.2676 23975157 PMC3819121

[B26] Magoč T. Salzberg S. L. (2011). FLASH: fast length adjustment of short reads to improve genome assemblies. *Bioinformatics* 27 2957–2963. 10.1093/bioinformatics/btr507 21903629 PMC3198573

[B27] Martiny J. B. Jones S. E. Lennon J. T. Martiny A. C. (2015). Microbiomes in light of traits: a phylogenetic perspective. *Science* 350:aac9323. 10.1126/science.aac9323 26542581

[B28] Morgan X. C. Tickle T. L. Sokol H. Gevers D. Devaney K. L. Ward D. V. (2012). Dysfunction of the intestinal microbiome in inflammatory bowel disease and treatment. *Genome Biol.* 13:R79. 10.1186/gb-2012-13-9-r79 23013615 PMC3506950

[B29] Nadal I. Donat E. Ribes-Koninckx C. Calabuig M. Sanz Y. (2007). Imbalance in the composition of the duodenal microbiota of children with coeliac disease. *J. Med. Microbiol.* 56 1669–1674. 10.1099/jmm.0.47410-0 18033837

[B30] Nicholson J. K. Holmes E. Wilson I. D. (2005). Gut microorganisms, mammalian metabolism and personalized health care. *Nat. Rev. Microbiol.* 3 431–438. 10.1038/nrmicro1152 15821725

[B31] O’Brien C. L. Allison G. E. Grimpen F. Pavli P. (2013). Impact of colonoscopy bowel preparation on intestinal microbiota. *PLoS One* 8:e62815. 10.1371/journal.pone.0062815 23650530 PMC3641102

[B32] Olesen J. M. Bascompte J. Dupont Y. L. Jordano P. (2007). The modularity of pollination networks. *Proc. Natl. Acad. Sci. U.S.A.* 104 19891–19896. 10.1073/pnas.0706375104 18056808 PMC2148393

[B33] Parks D. H. Tyson G. W. Hugenholtz P. Beiko R. G. (2014). STAMP: statistical analysis of taxonomic and functional profiles. *Bioinformatics* 30 3123–3124. 10.1093/bioinformatics/btu494 25061070 PMC4609014

[B34] Ryuk J. P. Choi G. S. Park J. S. Kim H. J. Park S. Y. Yoon G. S. (2014). Predictive factors and the prognosis of recurrence of colorectal cancer within 2 years after curative resection. *Ann. Surg. Treat. Res.* 86 143–151. 10.4174/astr.2014.86.3.143 24761423 PMC3994626

[B35] Saleh M. Trinchieri G. (2011). Innate immune mechanisms of colitis and colitis-associated colorectal cancer. *Nat. Rev. Immunol.* 11 9–20. 10.1038/nri2891 21151034

[B36] Scanlan P. D. Shanahan F. Marchesi J. R. (2009). Culture-independent analysis of desulfovibrios in the human distal colon of healthy, colorectal cancer and polypectomized individuals. *FEMS Microbiol. Ecol.* 69 213–221. 10.1111/j.1574-6941.2009.00709.x 19496818

[B37] Scharlau D. Borowicki A. Habermann N. Hofmann T. Klenow S. Miene C. (2009). Mechanisms of primary cancer prevention by butyrate and other products formed during gut flora-mediated fermentation of dietary fibre. *Mutat. Res.* 682 39–53. 10.1016/j.mrrev.2009.04.001 19383551

[B38] Shah M. S. Desantis T. Z. Weinmaier T. Mcmurdie P. J. Cope J. L. Altrichter A. (2017). Leveraging sequence-based faecal microbial community survey data to identify a composite biomarker for colorectal cancer. *Gut* 67 882–891. 10.1136/gutjnl-2016-313189 28341746

[B39] Shin N. R. Whon T. W. Bae J. W. (2015). *Proteobacteria*: microbial signature of dysbiosis in gut microbiota. *Trends Biotechnol.* 33 496–503. 10.1016/j.tibtech.2015.06.011 26210164

[B40] Sobhani I. Sobhani J. Roudot-Thoraval F. Roperch J. P. Letulle S. Letulle P. (2011). Microbial dysbiosis in colorectal cancer (CRC) patients. *PLoS One* 6:e16393. 10.1371/journal.pone.0016393 21297998 PMC3029306

[B41] Sun F. Wu D. Qiu Z. Jin M. Wang X. Li J. (2010). Development of real-time PCR systems based on SYBR Green for the specific detection and quantification of *Klebsiella pneumoniae* in infant formula. *Food Control* 21 487–491. 10.1016/j.foodcont.2009.07.014

[B42] Sun J. Kato I. (2016). Gut microbiota, inflammation and colorectal cancer. *Annu. Rev. Microbiol.* 3 130–143. 10.1016/j.gendis.2016.03.004 28078319 PMC5221561

[B43] Wang Q. Garrity G. M. Tiedje J. M. Cole J. R. (2007). Naïve Bayesian classifier for rapid assignment of rRNA sequences into the new bacterial taxonomy. *Appl. Environ. Microbiol.* 73 5261–5267. 10.1128/AEM.00062-07 17586664 PMC1950982

[B44] Willing B. P. Dicksved J. Halfvarson J. Andersson A. F. Lucio M. Zheng Z. (2010). A pyrosequencing study in twins shows that gastrointestinal microbial profiles vary with inflammatory bowel disease phenotypes. *Gastroenterology* 139 1844–1854. 10.1053/j.gastro.2010.08.049 20816835

[B45] Wu X. Zhang H. Chen J. Shang S. Wei Q. Yan J. (2016). Comparison of the fecal microbiota of dholes high-throughput Illumina sequencing of the V3–V4 region of the 16S rRNA gene. *Appl. Microbiol. Biotechnol.* 100 3577–3586. 10.1007/s00253-015-7257-y 26728019

[B46] Xiao Y. Liu X. Meng D. Tao J. Gu Y. Yin H. (2018). The role of soil bacterial community during winter fallow period in the incidence of tobacco bacterial wilt disease. *Appl. Microbiol. Biotechnol.* 102 2399–2412. 10.1007/s00253-018-8757-3 29368216

[B47] Yan Q. Gu Y. Li X. Yang W. Jia L. Chen C. (2017). Alterations of the gut microbiome in hypertension. *Front. Cell. Infect. Microbiol.* 7:381 10.3389/fcimb.2017.00381 PMC5573791 28884091

[B48] Zackular J. P. Rogers M. A. Schloss P. D. (2014). The human gut microbiome as a screening tool for colorectal cancer. *Cancer Prev. Res.* 7 1112–1121. 10.1158/1940-6207.CAPR-14-0129 25104642 PMC4221363

[B49] Zeller G. Tap J. Voigt A. Y. Sunagawa S. Kultima J. R. Costea P. I. (2014). Potential of fecal microbiota for early-stage detection of colorectal cancer. *Mol. Syst. Biol.* 10:766. 10.15252/msb.20145645 25432777 PMC4299606

[B50] Zheng Z. Zhong W. Liu L. Wu C. Zhang L. Cai S. (2016). Bioinformatics approaches for human gut microbiome research. *Infect. Dis. Transl. Med.* 2 69–79.

