# Supplementary material for: A Pilot Study: Changes of Gut Microbiota in Post-surgery Colorectal Cancer Patients
Source: Front Microbiol. 2018 Nov 20;9:2777. doi: 10.3389/fmicb.2018.02777 (PMC6255893; doi:10.3389/fmicb.2018.02777)
Supplement: Supplementary file 1 [file Table_1.DOCX]

Table S1 Clinicopathological variables in CRC patients

| Patients^a^ | TNM  Stage^b^ | Grade of Differentiation | Postoperative  fistula | Lymphatic invasion | Perineural invasion | Number of metastasis lymph nodes | Tumor marker | |
| --- | --- | --- | --- | --- | --- | --- | --- | --- |
|  |  |  |  |  |  |  | CEA(A0/A1) | CA199(A0/A1) |
| A-1 | III | Moderate | Yes | No | No | 2/33 | 2.15/2.44 | 4.25/- |
| A-2 | II | Moderate | Yes | No | No | 0/20 | -/- | -/- |
| A-3 | II | Moderate | Yes | No | No | 0/14 | 0.98/- | 3.89/- |
| A-4 | IV | Moderate-low | Yes | Yes | Yes | 9/16 | 1.35/1.33 | ＜0.60/＜0.60 |
| A-5 | II | Moderate | Yes | No | No | 0/11 | 14/- | ＜0.60/- |
| A-6 | III | Moderate | Yes | No | Yes | 2/11 | 1.28/0.70 | 16.80/11.72 |
| A-7 | III | Moderate | No | No | Yes | 1/18 | 9.59/3.67 | 17.36/11.72 |
| A-8 | III | low | Yes | Yes | Yes | 4/14 | 9.76/0.98 | 5.19/35.31 |
| A-9 | III | Moderate | Yes | No | No | 8/11 | 2.26/1.22 | 11.50/2.53 |
| A-10 | II | Moderate | No | No | Yes | 0/23 | 10.7/- | 14.59/7.81 |

^a^ Disease status confirmed by surgical pathology; ^b^ NCCN staging; CEA: carcino-embryonic antigen, reference ranges: 0-3.4; CA199: carbohydrate antigen 199, reference ranges: 0-39.
