# Supplementary material for: A Pilot Study: Changes of Gut Microbiota in Post-surgery Colorectal Cancer Patients
Source: Front Microbiol. 2018 Nov 20;9:2777. doi: 10.3389/fmicb.2018.02777 (PMC6255893; doi:10.3389/fmicb.2018.02777)
Supplement: Supplementary file 2 [file Table_2.DOCX]

Table S2 Detailed characteristics in healthy individuals and colorectal cancer patients

| Study subjects | ID | Sex | Age | BMI | Tumor location | Bowel treatment |
| --- | --- | --- | --- | --- | --- | --- |
| Colorectal cancer patients | A-1 | Female | 63 | 26.8 | rectum | Dixon |
|  | A-2 | Male | 62 | 25.1 | rectum | Dixon |
|  | A-3 | Male | 55 | 22.0 | rectum | Miles |
|  | A-4 | Male | 58 | 22.7 | rectum | Miles |
|  | A-5 | Male | 57 | 31.8 | rectum | Miles |
|  | A-6 | Male | 62 | 25.8 | rectum | Dixon |
|  | A-7 | Female | 34 | 19.9 | rectum | Dixon |
|  | A-8 | Female | 61 | 27.8 | rectum | Hartmann |
|  | A-9 | Male | 60 | 26.1 | rectum | Dixon |
|  | A-10 | Female | 54 | 22.0 | rectum | Dixon |
| Healthy individuals | H-1 | Female | 61 | 21.4 | / | / |
|  | H-2 | Female | 57 | 24.1 | / | / |
|  | H-3 | Male | 64 | 23.2 | / | / |
|  | H-4 | Male | 62 | 24.9 | / | / |
|  | H-5 | Female | 60 | 27.4 | / | / |
|  | H-6 | Female | 58 | 21.6 | / | / |
|  | H-7 | Female | 54 | 24.5 | / | / |
|  | H-8 | Female | 62 | 28.2 | / | / |
|  | H-9 | Female | 60 | 23.2 | / | / |
|  | H-10 | Female | 54 | 25.7 | / | / |
|  | H-11 | Female | 49 | 22.3 | / | / |
