# Supplementary material for: A Pilot Study: Changes of Gut Microbiota in Post-surgery Colorectal Cancer Patients
Source: Front Microbiol. 2018 Nov 20;9:2777. doi: 10.3389/fmicb.2018.02777 (PMC6255893; doi:10.3389/fmicb.2018.02777)
Supplement: Supplementary file 3 [file Table_3.DOCX]

Table S3 Three specific primers for gut microbiota in real-time qPCR

| Primers | Sequence (5’-3’) | Fragment length (bases) | Annealing Temperature (℃) | Reference |
| --- | --- | --- | --- | --- |
| Bacteria | 338F: ACTCCTACGGGAGGCAGCAG  518R: ATTACCGCGGCTGCTGG | 197 | 57 | [Ovreås et al., 1997](#_ENREF_2) |
| *Fusobacterium nucleatum* | F: CAACCATTACTTTAACTCTACCATGTTCA  R:GTTGACTTTACAGAAGGAGATTATGTAAAAATC | 161 | 57 | [Castellarin et al., 2012](#_ENREF_1) |
| *Klebsiella pneumoniae* | F: TGCCCAGACCGATAACTTTA  R: CTGTTTCTTCGCTTCACGG | 142 | 57 | [Sun et al., 2010](#_ENREF_3) |

Ovreås, L., Forney, L., Daae, F.L., and Torsvik, V. (1997). Distribution of bacterioplankton in meromictic Lake Saelenvannet, as determined by denaturing gradient gel electrophoresis of PCR-amplified gene fragments coding for 16S rRNA. Applied & Environmental Microbiology 63(9), 3367.

Castellarin, M., Warren, R.L., Freeman, J.D., Dreolini, L., Krzywinski, M., Strauss, J., et al. (2012). Fusobacterium nucleatum infection is prevalent in human colorectal carcinoma. Genome Research 22(2), 299-306.

Sun, F., Wu, D., Qiu, Z., Jin, M., Wang, X., and Li, J. (2010). Development of real-time PCR systems based on SYBR Green for the specific detection and quantification of Klebsiella pneumoniae in infant formula. Food Control 21(4), 487-491.
