# Supplementary material for: A Pilot Study: Changes of Gut Microbiota in Post-surgery Colorectal Cancer Patients
Source: Front Microbiol. 2018 Nov 20;9:2777. doi: 10.3389/fmicb.2018.02777 (PMC6255893; doi:10.3389/fmicb.2018.02777)
Supplement: Supplementary file 4 [file Table_4.DOCX]

Table S4 Numbers of clean reads and number of OTUs (97% similarity level) in each sample

| Healthy individuals | | | Colorectal cancer patients | | | | | |
| --- | --- | --- | --- | --- | --- | --- | --- | --- |
|  |  |  | Pro-surgery | | | Post-surgery | | |
| Sample name | Clean reads | OTUs (97%) | Sample name | Clean reads | OTUs (97%) | Sample name | Clean reads | OTUs (97%) |
| H-1 | 56107 | 122 | A0-1 | 57576 | 242 | A1-1 | 58582 | 223 |
| H-2 | 59379 | 209 | A0-2 | 55944 | 195 | A1-2 | 62618 | 288 |
| H-3 | 58286 | 192 | A0-3 | 55946 | 125 | A1-3 | 55869 | 146 |
| H-4 | 55334 | 200 | A0-4 | 58630 | 204 | A1-4 | 59444 | 108 |
| H-5 | 63624 | 221 | A0-5 | 56628 | 136 | A1-5 | 58089 | 134 |
| H-6 | 57153 | 126 | A0-6 | 57683 | 220 | A1-6 | 56118 | 153 |
| H-7 | 58475 | 211 | A0-7 | 60531 | 254 | A1-7 | 55763 | 94 |
| H-8 | 57454 | 251 | A0-8 | 61209 | 161 | A1-8 | 60654 | 178 |
| H-9 | 58290 | 213 | A0-9 | 56762 | 183 | A1-9 | 64206 | 87 |
| H-10 | 61579 | 146 | A0-10 | 63769 | 319 | A1-10 | 61323 | 315 |
| H-11 | 56185 | 217 |  |  |  |  |  |  |
