# Supplementary material for: A Pilot Study: Changes of Gut Microbiota in Post-surgery Colorectal Cancer Patients
Source: Front Microbiol. 2018 Nov 20;9:2777. doi: 10.3389/fmicb.2018.02777 (PMC6255893; doi:10.3389/fmicb.2018.02777)
Supplement: Supplementary file 5 [file Table_5.DOCX]

Table S5 Statistical analysis of differences in the gut microbial communities among H, A0 and A1

| Sample | A1 VS A0 | A1 VS H | A0 VS H |
| --- | --- | --- | --- |
| MRPP (A/P value) | **0.075/0.01** | **0.090/0.004** | -0.016/0.908 |
| Anosim (R/P value) | **0.227/0.016** | **0.302/0.001** | -0.06/0.901 |
