# Supplementary material for: A Pilot Study: Changes of Gut Microbiota in Post-surgery Colorectal Cancer Patients
Source: Front Microbiol. 2018 Nov 20;9:2777. doi: 10.3389/fmicb.2018.02777 (PMC6255893; doi:10.3389/fmicb.2018.02777)
Supplement: Supplementary file 6 [file Table_6.DOCX]

Table S6 Comparisons of healthy individuals and CRC patients based on the relative abundance at the phylum level

| Phylum | H(%) | A0(%) | A1(%) | *P*(H-A0) | *P*(H-A1) | *P*(A0-A1) |
| --- | --- | --- | --- | --- | --- | --- |
| *Firmicutes* | 47.71 | 45.76 | 55.46 | 0.822 | 0.374 | 0.279 |
| *Bacteroidetes* | 48.24 | 48.09 | 25.18 | 0.986 | **0.012** | **0.015** |
| *Proteobacteria* | 3.15 | 3.94 | 16.05 | 0.882 | **0.022** | **0.034** |
| *Actinobacteria* | 0.46 | 1.06 | 2.10 | 0.532 | 0.098 | 0.300 |
| *Fusobacteria* | 0.30 | 1.10 | 0.79 | 0.164 | 0.390 | 0.592 |
| *Candidatus Saccharibacteria* | 0.00 | 0.01 | 0.35 | 0.968 | 0.101 | 0.117 |
| *Verrucomicrobia* | 0.09 | 0.01 | 0.01 | 0.276 | 0.285 | 0.983 |
| *Euryarchaeota* | 0.05 | 0.02 | 0.04 | 0.567 | 0.777 | 0.776 |
