# Supplementary material for: A Pilot Study: Changes of Gut Microbiota in Post-surgery Colorectal Cancer Patients
Source: Front Microbiol. 2018 Nov 20;9:2777. doi: 10.3389/fmicb.2018.02777 (PMC6255893; doi:10.3389/fmicb.2018.02777)
Supplement: Supplementary file 7 [file Table_7.DOCX]

Table S7 Comparisons of healthy individuals and CRC patients based on the relative abundance at the genus level

| Genus | H(%) | A0(%) | A1(%) | *P*(H-A0) | *P*(H-A1) | *P*(A0-A1) |
| --- | --- | --- | --- | --- | --- | --- |
| *Bacteroides* | 40.53 | 38.18 | 22.68 | 0.811 | 0.078 | 0.132 |
| *Faecalibacterium* | 10.00 | 7.26 | 3.13 | 0.305 | **0.014** | 0.136 |
| *Prevotella* | 6.64 | 8.54 | 1.28 | 0.767 | 0.407 | 0.274 |
| *Roseburia* | 7.59 | 7.29 | 1.09 | 0.910 | **0.020** | **0.029** |
| *Enterococcus* | 0.00 | 0.02 | 13.51 | 0.998 | 0.087 | 0.094 |
| *Dialister* | 3.32 | 4.47 | 5.07 | 0.781 | 0.673 | 0.888 |
| *Escherichia/Shigella* | 0.35 | 1.73 | 5.70 | 0.655 | 0.091 | 0.214 |
| *Klebsiella* | 0.62 | 0.95 | 5.63 | 0.897 | 0.058 | 0.083 |
| *Clostridium XlVa* | 2.95 | 3.39 | 1.76 | 0.693 | 0.287 | 0.158 |
| *Veillonella* | 0.64 | 1.62 | 4.35 | 0.742 | 0.216 | 0.370 |
| *Ruminococcus* | 5.15 | 1.50 | 0.25 | **0.040** | **0.007** | 0.478 |
| *Clostridium sensu stricto* | 0.15 | 0.07 | 6.21 | 0.988 | 0.206 | 0.211 |
| *Parabacteroides* | 1.52 | 2.31 | 2.41 | 0.614 | 0.570 | 0.949 |
| *Phascolarctobacterium* | 2.17 | 1.29 | 2.54 | 0.548 | 0.804 | 0.408 |
| *Haemophilus* | 1.47 | 0.15 | 3.01 | 0.551 | 0.487 | 0.212 |
| *Ruminococcus2* | 1.52 | 2.31 | 0.88 | 0.360 | 0.458 | 0.111 |
| *Alistipes* | 1.76 | 2.26 | 0.45 | 0.491 | 0.081 | **0.021** |
| *Lachnospiracea_incertae_sedis* | 1.88 | 1.03 | 0.56 | 0.199 | **0.050** | 0.481 |
| *Gemmiger* | 1.42 | 1.34 | 0.62 | 0.922 | 0.284 | 0.340 |
| *Lactobacillus* | 0.02 | 0.03 | 2.72 | 0.997 | 0.186 | 0.197 |
| Other | 10.31 | 14.26 | 16.15 | 0.268 | 0.106 | 0.602 |

*P* was calculated by ANOVA
