# Supplementary material for: A Pilot Study: Changes of Gut Microbiota in Post-surgery Colorectal Cancer Patients
Source: Front Microbiol. 2018 Nov 20;9:2777. doi: 10.3389/fmicb.2018.02777 (PMC6255893; doi:10.3389/fmicb.2018.02777)
Supplement: Supplementary file 8 [file Table_8.DOCX]

Table S8 Relationships between gut microbiota and environmental variables in CRC patients based on the mantel test.

| Gut microbiota | Age  (*P* value-A0/  *P* value-A1) | Sex  (*P* value-A0/  *P* value-A1A1) | BMI  (*P* value-A0/  *P* value-A1A1) | Bowel treatment  (*P* value-A1) |
| --- | --- | --- | --- | --- |
| The whole | 0.625/0.301 | 0.069/0.413 | 0.220/0.906 | 0.840 |
| *Actinobacteria* | 0.405/0.810 | 0.231/0.802 | 0.610/0.557 | 0.243 |
| *Bacteroidetes* | 0.300/0.078 | 0.073/0.324 | 0.203/0.085 | 0.222 |
| *Firmicutes* | 0.855/0.724 | 0.822/0.500 | 0.820/0.950 | 0.862 |
| *Fusobacteria* | 0.74/0.051 | 0.831/0.243 | 0.254/0.259 | 0.964 |
| *Proteobacteria* | 0.581/0.649 | 0.344/0.708 | 0.509/0.991 | 0.986 |
| *Verrucomicrobia* | 0.243/0.368 | 0.468/0.166 | 0.706/0.601 | 0.642 |
