# Supplementary material for: A Pilot Study: Changes of Gut Microbiota in Post-surgery Colorectal Cancer Patients
Source: Front Microbiol. 2018 Nov 20;9:2777. doi: 10.3389/fmicb.2018.02777 (PMC6255893; doi:10.3389/fmicb.2018.02777)
Supplement: Supplementary file 10 [file Data_Sheet_1.PDF]

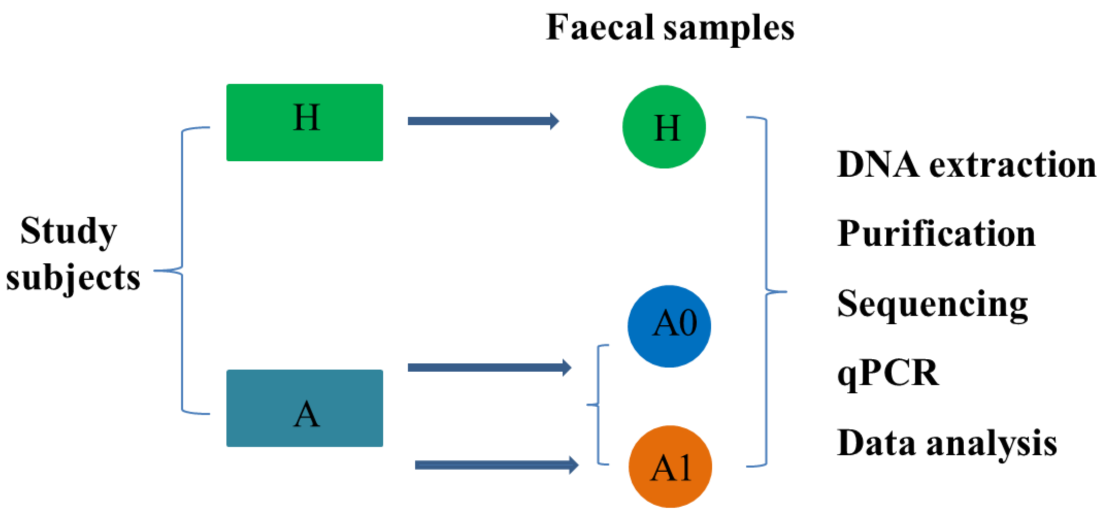

Figure S1 Study design. Eleven healthy individuals (H) and ten colorectal cancer patients (A) were recruited for the study (see Table 1 and Table S1). Fecal samples from these pre-surgery CRC patients (A0) were collected prior to a colonoscopy. All patients diagnosed with CRC were treated with palliative surgery or radical surgery, such as Dixon, Miles and Hartmann. These post-surgery samples (A1) were obtained in approximately one month after the surgery for colorectal cancer patients.

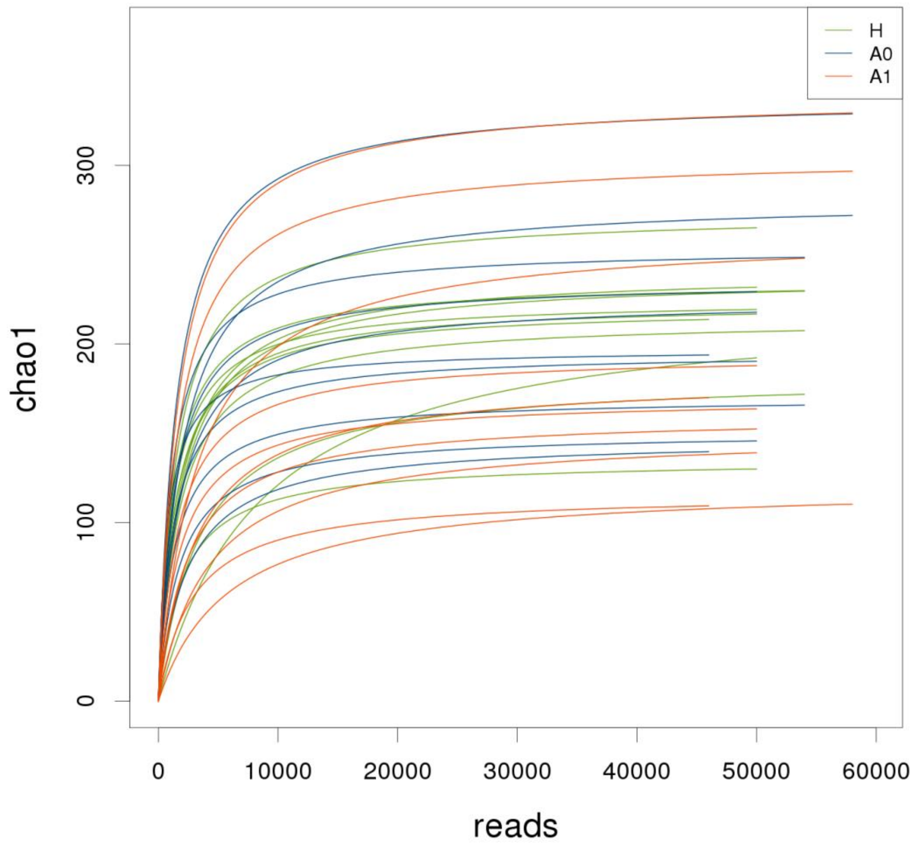

Figure S2 Rarefaction for CRC patients and healthy individuals was generated at 97% similarity level.

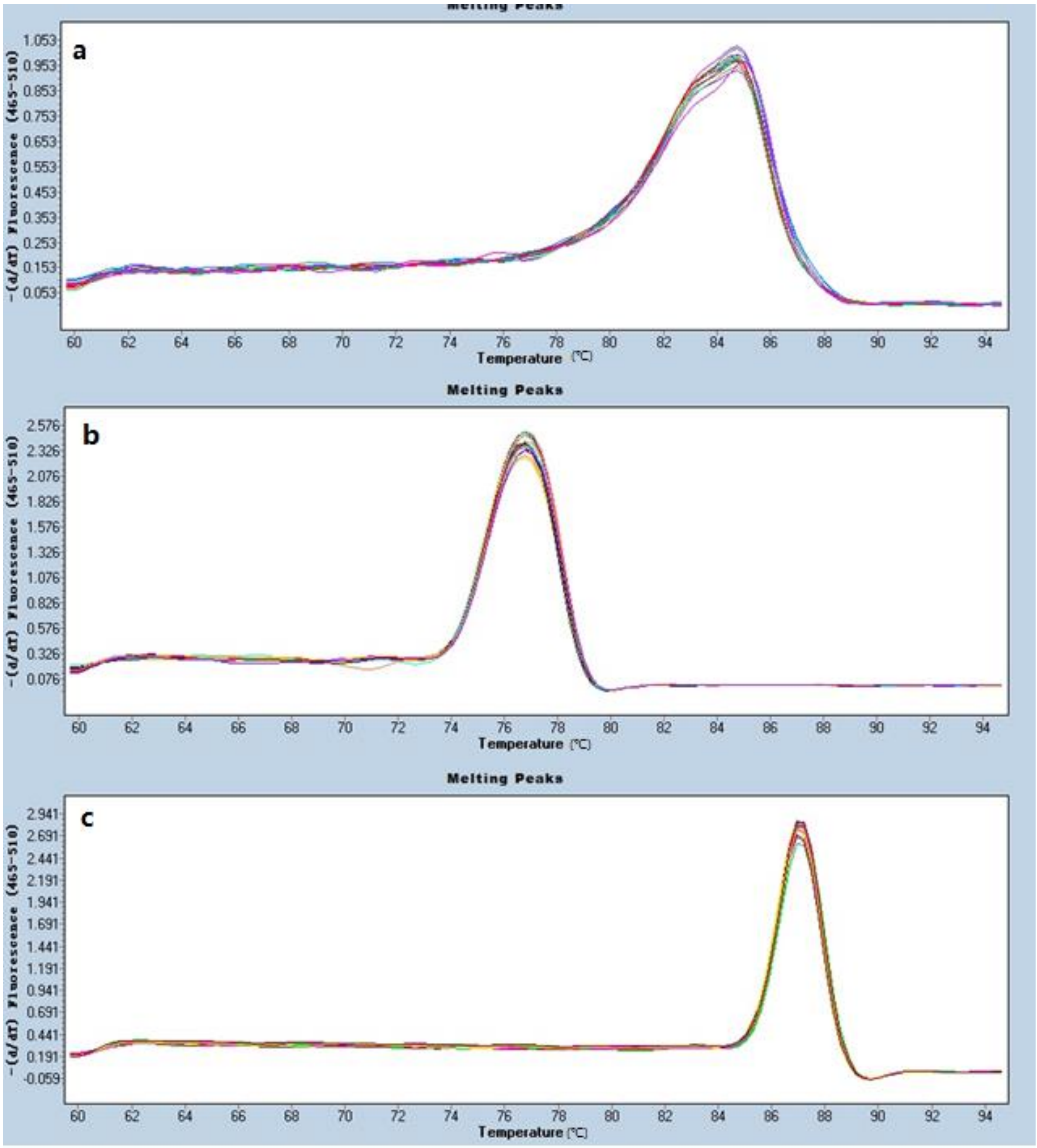

Figure S3 Melting curves of three primers during real-time qPCR. (a) 16S rRNA universal primer for *Bacteria*, (b) primer for species *Fusobacterium nucleatum*, (c) primer for species *Klebsiella pneumoniae*.

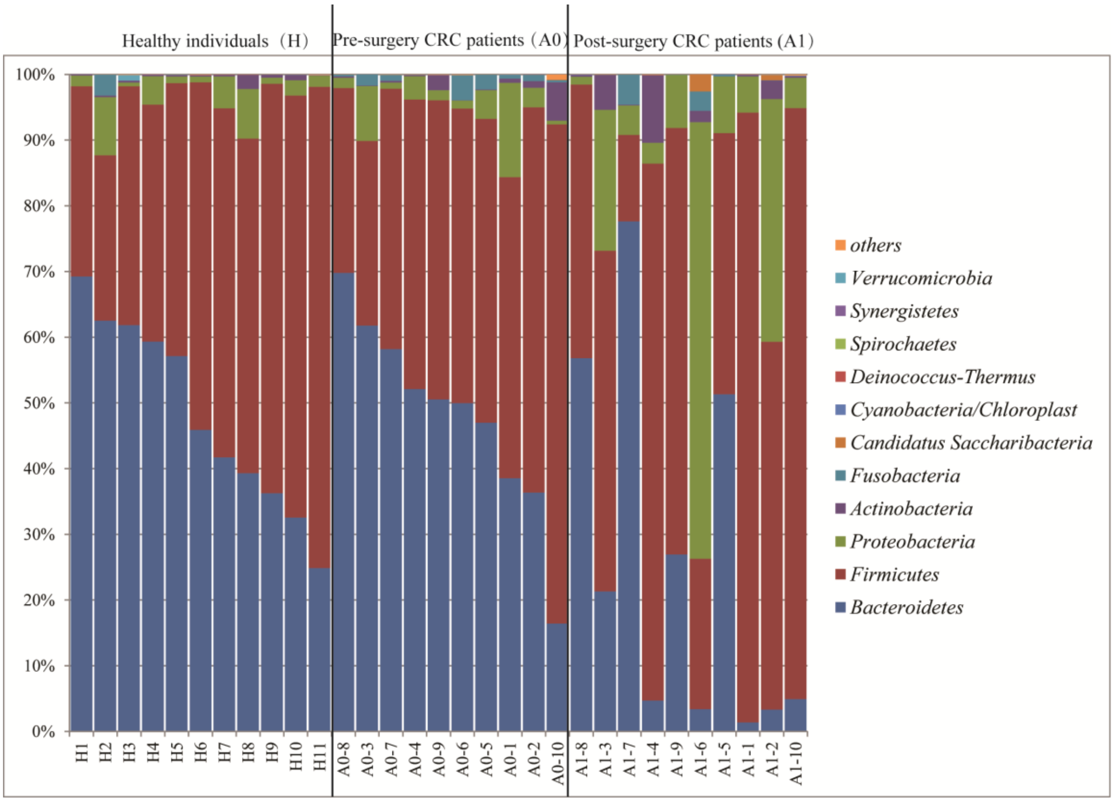

Figure S4 Taxonomic composition of gut microbiota at the phylum level in colorectal cancer patients and healthy individuals.

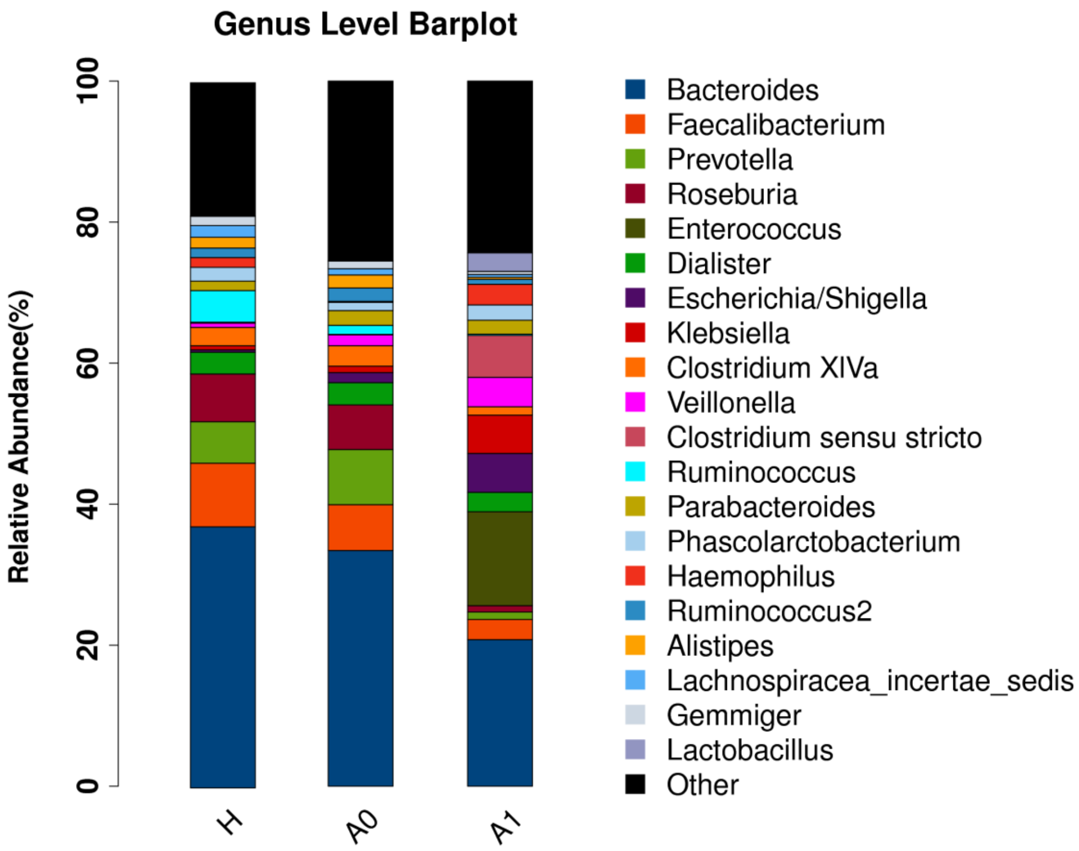

Figure S5 Taxonomic composition at the genus level of gut microbiota.

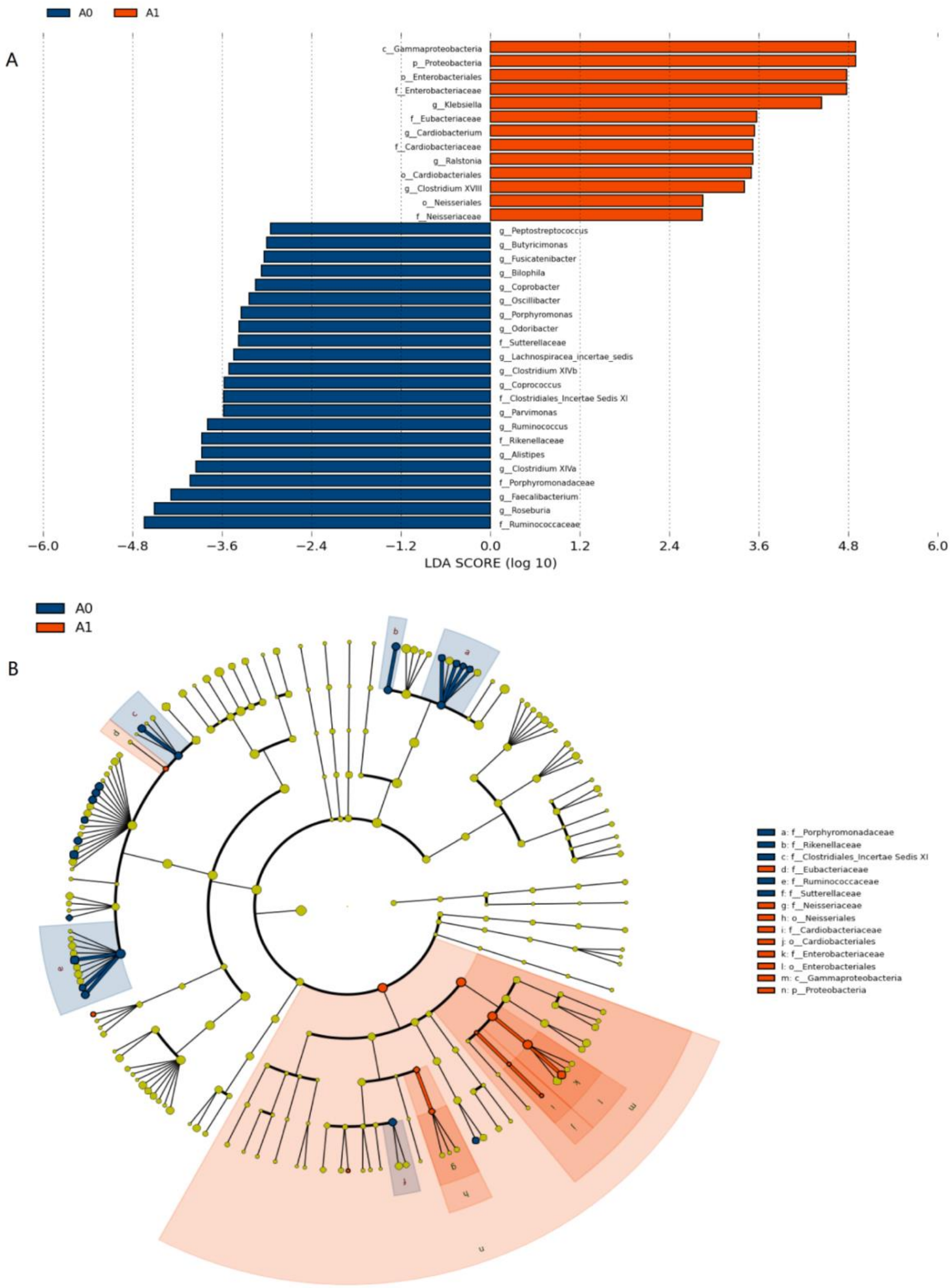

Figure S6 Microbial biomarkers among healthy volunteers (H) and CRC patients (A0 and A1). **A.** LEfSe analysis shows differentially abundant taxa as biomarkers using Kruskal-Wallis test ($P < 0.05$) with LDA score > 2.0. **B.** Cladogram representation of the differentially abundant taxa. The root of the cladogram represents the domain bacteria. The size of each node represents their relative abundance. No significantly different taxa were labeled by yellow. Significant different taxa were labeled by following the color of each group.

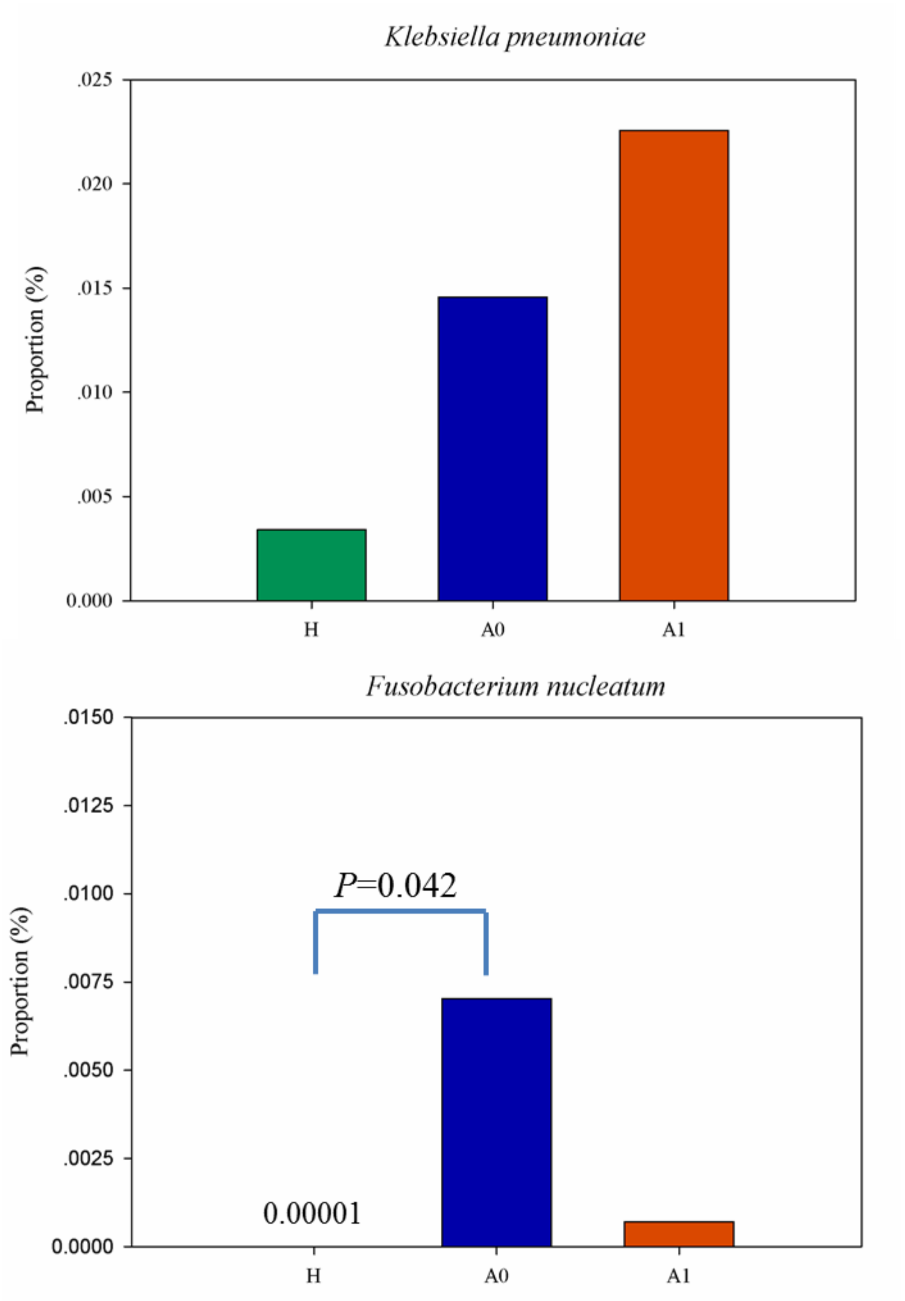

Figure S7 The relative abundance of *Klebsiella pneumoniae* and *Fusobacterium nucleatum* based on real time qPCR.

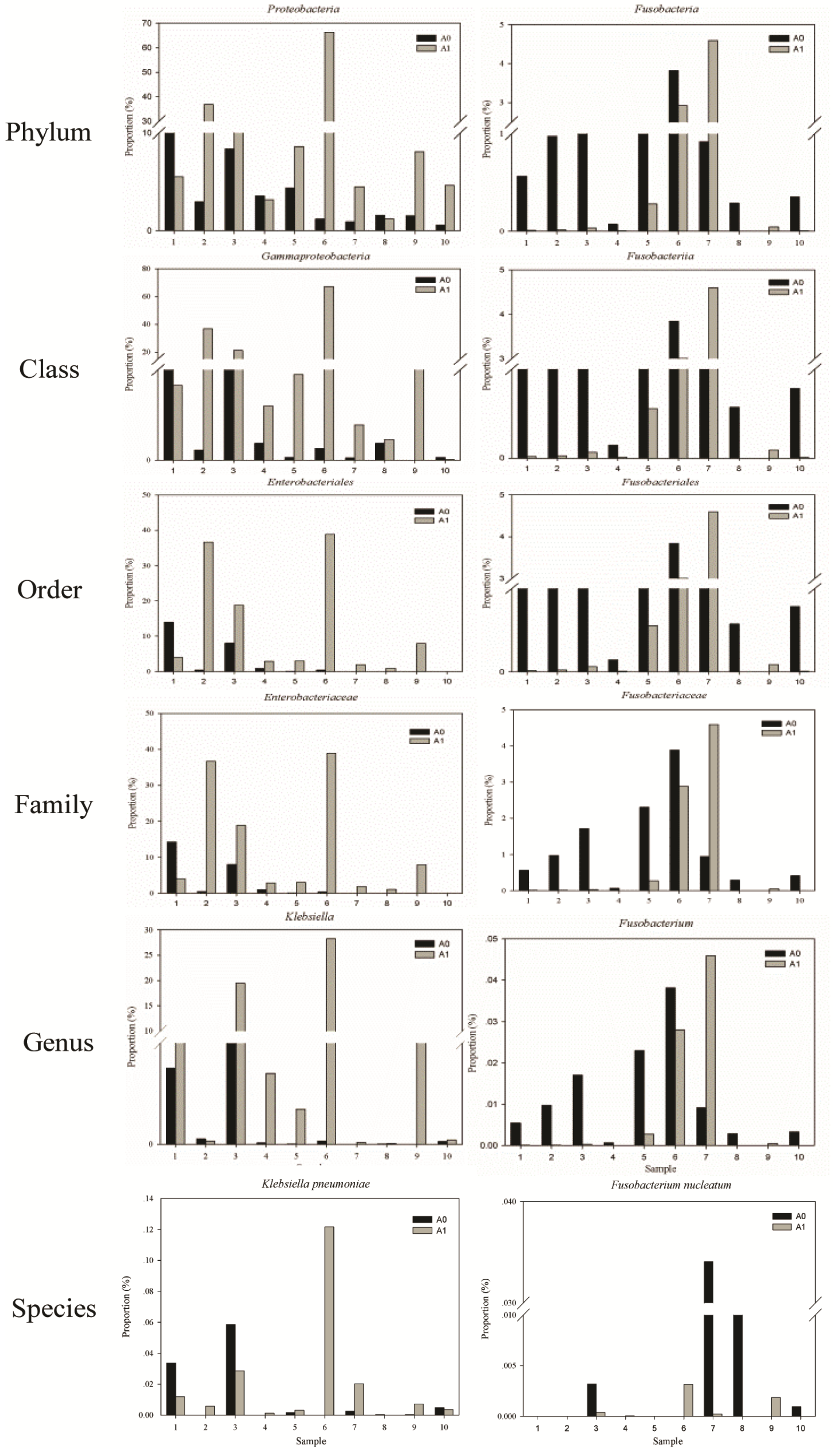

Figure S8 Changes of key gut microbiota in CRC patients in response to surgery.

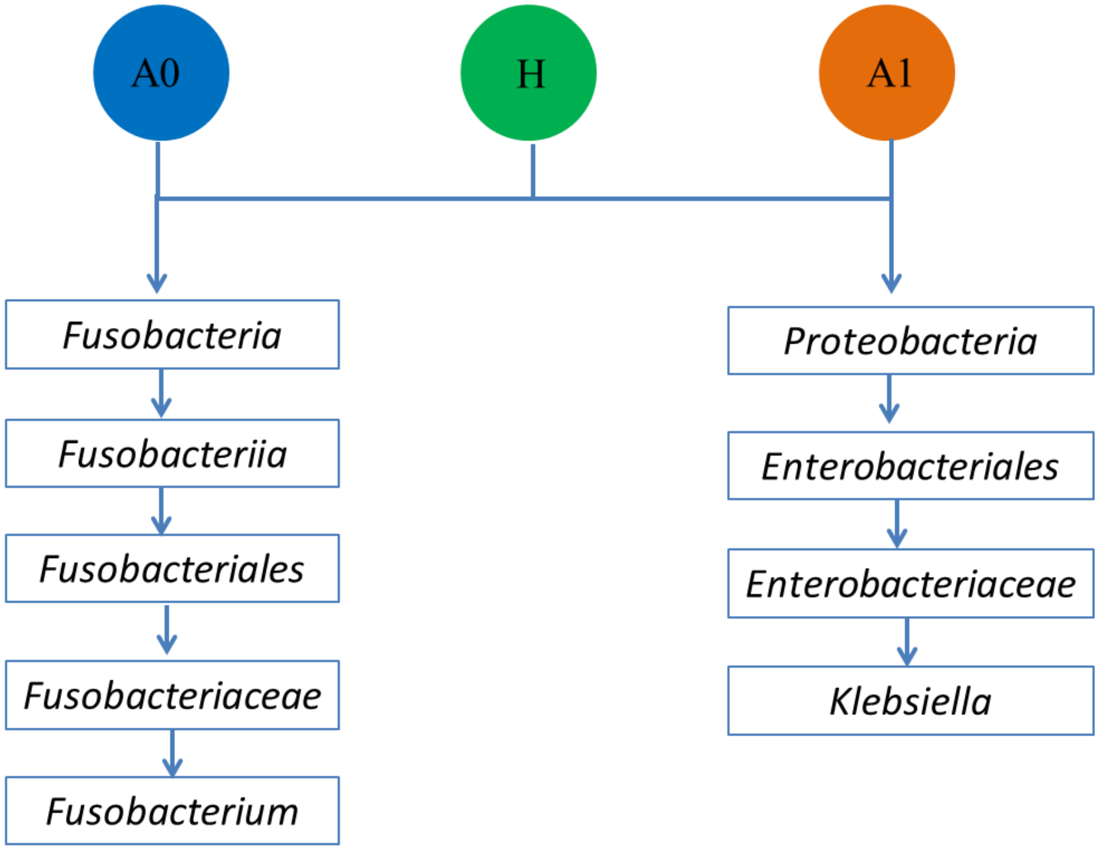

Figure S9 Main significantly different microbes between healthy individuals and colorectal cancer patients before and after the surgery
